# Somewhere to go: assessing the impact of public restroom interventions on reports of open defecation in San Francisco, California from 2014 to 2020

**DOI:** 10.1186/s12889-022-13904-4

**Published:** 2022-09-04

**Authors:** Heather K. Amato, Douglas Martin, Christopher M. Hoover, Jay P. Graham

**Affiliations:** 1 Division of Environmental Health Sciences, Berkeley School of Public Health, University of California, Berkeley, USA; 2 Berkeley School of Public Health, University of California, Berkeley, USA

**Keywords:** Open defecation, Environmental contamination, Sanitation, San Francisco, Public toilets, Homelessness

## Abstract

**Background:**

Open defecation due to a lack of access to sanitation facilities remains a public health issue in the United States. People experiencing homelessness face barriers to accessing sanitation facilities, and are often forced to practice open defecation on streets and sidewalks. Exposed feces may contain harmful pathogens posing a significant threat to public health, especially among unhoused persons living near open defecation sites. The City of San Francisco’s Department of Public Works implemented the Pit Stop Program to provide the unhoused and the general public with improved access to sanitation with the goal of reducing fecal contamination on streets and sidewalks. The objective of this study was to assess the impact of these public restroom interventions on reports of exposed feces in San Francisco, California.

**Methods:**

We evaluated the impact of various public restroom interventions implemented from January 1, 2014 to January 1, 2020 on reports of exposed feces, captured through a 311 municipal service. Publicly available 311 reports of exposed feces were spatially and temporally matched to 31 Pit Stop restroom interventions at 27 locations across 10 San Francisco neighborhoods. We conducted an interrupted time-series analysis to compare pre- versus post-intervention rates of feces reports near the restrooms.

**Results:**

Feces reports declined by 12.47 reports per week after the installation of 13 Pit Stop restrooms (*p-*value = 0.0002). In the same restrooms, the rate of reports per week declined from the six-month pre-intervention period to the post-intervention period (slope change = -0.024 [95% CI = -0.033, -0.014]). In a subset of restrooms, where new installations were made (Mission and Golden Gate Park), and in another subset of restrooms where restroom attendants were provided (Mission, Castro/Upper Market, and Financial District/South Beach), feces reports also declined.

**Conclusions:**

Increased access to public toilets reduced feces reports in San Francisco, especially in neighborhoods with people experiencing homelessness. The addition of restroom attendants also appeared to have reduced feces reports in some neighborhoods with PEH. These interventions should be audited for implementation quality, observed utilization data, and user experience at the neighborhood level in order to tailor sanitation interventions to neighborhood-specific needs.

**Supplementary Information:**

The online version contains supplementary material available at 10.1186/s12889-022-13904-4.

## Background

Open defecation in several neighborhoods of San Francisco, California has been highlighted as a problem in recent years [1]. However, no rigorous studies have been conducted to understand how access to public restrooms can potentially mitigate open defecation in this setting. A recent study by Capone et al. suggested that at least 930,000 individuals in the United States lack access to basic sanitation, in striking contrast to previous estimates by the World Health Organization Joint Monitoring Program (JMP) which placed the number at 28,000 [2]. Critically, Capone’s estimate was the first to include people experiencing homelessness (PEH), who collectively accounted for approximately half of the population lacking access to basic sanitation (460,000). Notably, the JMP has reported that open defecation (disposal of human feces in fields, forests, bushes, open bodies of water, beaches, and other open spaces) and limited sanitation (use of improved facilities shared between two or more households) are nonexistent in the United States [3]. Capone et al. argues that all unsheltered PEH should be classified as engaging in open defecation and that most sheltered PEH should be classified as having limited sanitation [2]. In 2019, there were an estimated 8,000 PEH in San Francisco, 64% of which were unsheltered [4].

Unsheltered PEH rely on public restrooms, homeless service agencies, and privately owned business restrooms for their sanitation needs. Access to these facilities can be restricted by barriers such as limited hours of operation, transportation difficulties when traveling to distant facilities, customer-only policies at businesses, discrimination against PEH by staff members, and insufficient levels of cleanliness, maintenance, and monitoring [1, 5]. PEH may be forced to practice open defecation, which may have detrimental effects on their physical, mental, and social well-being. Open defecation also constitutes a public health hazard: exposure to fecal contamination in the environment can spread pathogenic infections causing diarrheal and other illnesses [6, 7]. Limited research has shown that homelessness may be a risk factor for certain infectious diseases in San Francisco [8, 9]. Unsheltered PEH may be most at risk of exposure to fecal pathogens if they occupy public sidewalks or other spaces where open defecation occurs and do not have reliable access to water or sanitation for hygiene practices.

Seeking to address these issues and reduce open defecation, the San Francisco Department of Public Works (DPW) began the Pit Stop Program in 2014. This program provides free, public restrooms throughout the city, many of which are staffed with two paid attendants. The attendants ensure the Pit Stop is clean, safe, and adequately stocked with supplies. All Pit Stops are also equipped with waste bins, dog waste bags, and needle disposal boxes. The DPW utilizes a citywide 311 municipal reporting system for individuals to report exposed feces found on public property. Using these reports as a proxy for instances of open defecation, we retrospectively investigated the impacts of different Pit Stop public restroom interventions on reports of exposed feces in San Francisco by analyzing the pre- versus post-intervention change in weekly feces reports near each restroom.

## Methods

### Pit stop restroom interventions

We evaluated the impact of San Francisco Pit Stop interventions implemented between January 1, 2014 and January 1, 2020. Information on Pit Stop locations and intervention start dates was provided by the San Francisco DPW upon request. Within the Pit Stop Program, we identified three categories of sanitation interventions: 1) installation of new restroom (the provision of portable staffed Pit Stop facilities in locations where no public restrooms previously existed); 2) provision of attendants (the conversion of previously existing unstaffed public restrooms into staffed Pit Stops); and 3) expansion of service hours (the extension of hours of operation from daytime-only to 24 h per day, a 2019 pilot program). Existing restrooms that were converted to staffed Pit Stops included self-cleaning JC Decaux facilities and Recreation and Park Department facilities. New portable Pit Stop restrooms have 1–2 gender-neutral units, each with a stall and sink. JC Decaux Pit Stop restrooms have a gender-neutral unit with a single stall and sink, which are automatically sanitized in between users. The Recreation and Park Department Pit Stop restrooms are public park restrooms (with separate units for men and women, each with 1–2 stalls, a sink, and a urinal in the men’s unit) which have been staffed with attendants. All Pit Stop restrooms are wheelchair accessible.

### Study site description

**Fig. 1:**
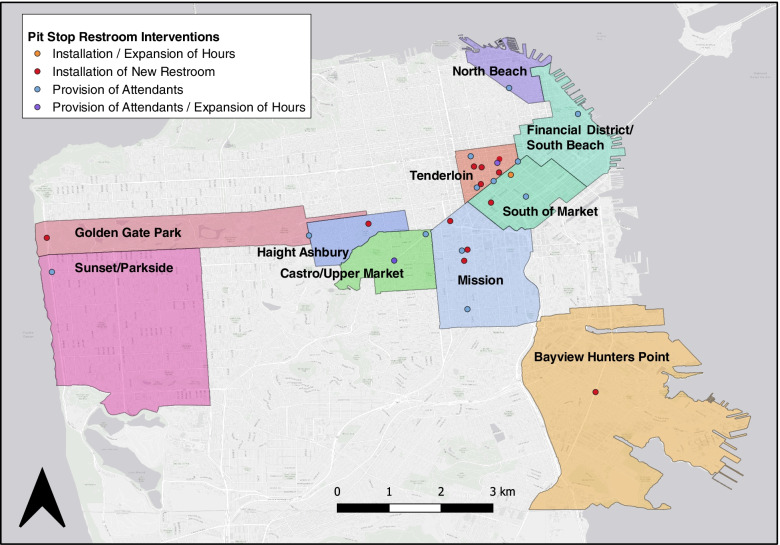
Restroom interventions implemented in San Francisco neighborhoods, 2014–2020. Legend: All 27 Pit Stop public restroom locations included in the analysis are shown on this map. Shaded and labeled areas represent neighborhoods. Orange and purple dots indicate multiple interventions occurred at a single Pit Stop restroom location. Map was generated using QGIS (version 3.12.1) with Pit Stop data provided by the SF DPW, neighborhood boundary shapefiles publicly available from https://data.sfgov.org/, and ESRI basemap data

There were ten neighborhoods that were involved in the San Francisco Pit Stop Program and were included in the analysis (Fig. 1). Neighborhood and district boundaries were defined by the San Francisco municipal government. In 2019, District 6, which contains the Tenderloin and SoMa neighborhoods, had an estimated 3,656 homeless residents. District 10 had the second highest number of homeless residents (1,820 PEH). Among these residents, over half were estimated to be unsheltered [4].

### Reports of exposed feces

San Francisco 311 reports since 2008 are publicly available at https://datasf.org/opendata/. The 311 reporting system includes a variety of municipal services and several report classification systems to route reports to appropriate agencies. Each report includes the report type (e.g. Human/Animal Waste), responsible agency, date, location (street address and longitude/latitude), status notes, and a photo of the incident (if provided by the individual making the report). Only reports of type Human/Animal Waste were included in this analysis, as these correspond to incidents of exposed feces. Human/Animal Waste were classified as a single report type, so human waste reports could not be distinguished from animal waste reports. To remove duplicate or misclassified reports, we excluded reports with “dup” or “transfer” in the status notes and only included reports from agencies that respond to feces reports. We consulted with DPW staff to help develop and validate these data processing methods.

### Spatial analysis

We mapped Pit Stop locations from GPS coordinates (provided by the San Francisco DPW) using QGIS Geographic Information System (version 3.12.1). For neighborhood boundaries, we used Analysis Neighborhoods polygons created by the San Francisco Department of Public Health, available here: https://data.sfgov.org/Geographic-Locations-and-Boundaries/Analysis-Neighborhoods/p5b7-5n3h. In ArcGIS Online (Esri), we created 500-m walking distance buffers (polygon derived from all 500 m routes following pedestrian paths and roads) around each Pit Stop location to capture the number of 311 feces reports within the surrounding area of each intervention. Feces reports were then spatially and temporally matched to each Pit Stop intervention in R version 4.0.2 [10] using the *data.table* and *sf*packages [11, 12]. We identified all 311 feces reports that occurred within a 500 m walking distance buffer of each intervention location and within six months before and six months after the intervention start date.

### Statistical methods

Our main outcome of interest was the number of exposed feces reports per week within 500 m of each Pit Stop location. We calculated the means and standard deviations (SD) of reports in a six-month period before the intervention and during the six months after the intervention by intervention type and neighborhood. We obtained *p*-values from permutation tests (*N*= 10,000 permutations) using an alpha of 0.05 to determine statistical significance of the difference in sample means [13]. We also calculated means and SDs of reports by year and season.

We used an interrupted time series approach to further analyze the longitudinal impacts of Pit Stop interventions on reports of exposed feces [14]. We assessed longitudinal trends in 311 feces reports per week during the six-month (26-week) period before versus the six-month period after each intervention. We specified the following negative binomial model, appropriate for modeling overdispersion in weekly count data: [15]

$$\mathrm{log}\left({\mathbb{E}}\left[{\mu }_{it}|{\varvec{X}}\right]\right)={\beta }_{0}+{\beta }_{1}Wee{k}_{t}+{\beta }_{2}Interventio{n}_{it}+ {\beta }_{3}Wee{k}_{t}Interventio{n}_{it}+ {\beta }_{4}Confounder$$

where ${\mu }_{it}$ is the mean number of 311 feces reports per week for a given Pit Stop $i$ in week $t$, and the *intervention* variable is binary variable indicating whether the intervention has been implemented at site $i$ at time $t$. We use ${\varvec{X}}$ as shorthand for the entire set of independent variables, which include the week, *t*, the intervention status at Pit Stop $i$ at time $t$ (pre-/post-intervention), and confounding variables identified a priori (described below)*.* This model accounts for a single time-point level change at the time of each intervention (${\beta }_{2}$), as well as a post-intervention slope change with the inclusion of an interaction term (${\beta }_{3}$) for the specific week and intervention status [14].

We estimated the change in the rate of feces reports per week (i.e. the post-intervention slope change) by intervention type, as well as by neighborhood and intervention type. We included neighborhood type in the models stratified by intervention type to adjust for confounding due to spatial dependence of Pit Stops within the same neighborhood. Sandwich estimators were used to calculate robust standard errors and 95% confidence intervals. To establish a transition period between the pre- and post-intervention samples, we removed the 27^th^ week (which included the intervention start date) for each Pit Stop intervention for all analyses. Statistical analyses were conducted in R version 4.0.2 [10] using the following packages: *dplyr*, *perm, Mass, lmtest,* and *sandwich* [16–19]. Plots were created using *ggplot2* and *ggpubr* packages [20, 21].

## Results

There were 31 Pit Stop interventions implemented across 27 locations within 10 neighborhoods between January 1, 2014 and January 1, 2020, including the installation of 13 new restrooms (Table 1; Fig. 1). Existing restrooms were staffed with attendants at 15 locations throughout the study period, and three restrooms expanded their service hours beginning in 2019. The earliest interventions included in the analysis were three new restrooms installed in the Tenderloin on July 15, 2014, and the most recent interventions were the expansion of service hours at three existing restrooms in different neighborhoods on August 16, 2019 (Supplemental Materials, Table S1). The number of exposed feces reports within a 500 m walking distance of each Pit Stop intervention ranged from 0–201 reports per week. During the six-year study period, the highest mean number of feces reports per week occurred in the spring (mean = 36.8, SD = 40.2), followed by summer (mean = 35.3, SD = 35.8), winter (mean = 29.6, SD = 29.4), and fall (mean = 28.4, SD = 21.6) (Figure S1).

**Table 1:** Pre- versus post-intervention mean feces reports per week by intervention type and neighborhood

	No. Pit Stop Interventions	No. Weeks Observed ^a^	Mean reports per week pre-intervention (SD)	Mean reports per week post-intervention (SD)	Change in Mean (Δ)	*p-*value ^b^
*Intervention Type*						
Installation of New Restroom	13	338	49.18 (48.45)	36.71 (27.17)	-12.47	0.0002
Provision of Attendants	15	390	22.75 (26.56)	20.87 (16.95)	-1.88	0.2296
Expansion of Service Hours	3	78	34.45 (16.92)	46.45 (29.45)	12	0.0016
*Neighborhood of Intervention*						
Tenderloin ^c^	11	286	68.01 (45.18)	50.40 (21.89)	-17.60	0.0002
Mission ^c^	5	130	26.98 (12.71)	28.24 (11.57)	1.25	0.4068
South of Market (SoMa) ^c^	4	104	37.42 (13.92)	39.38 (21.55)	1.95	0.4406
Castro/Upper Market	4	104	8.99 (5.30)	12.08 (6.19)	3.09	0.0004
Golden Gate Park ^c^	2	52	0.69 (1.04)	0.85 (1.23)	0.15	0.5561
Haight Ashbury ^c^	1	26	1.08 (1.38)	1.85 (1.91)	0.77	0.1242
Bayview Hunters Point ^c^	1	26	2.50 (1.48)	2.35 (1.72)	-0.15	0.7953
Sunset Parkside	1	26	0.27 (0.53)	0.65 (0.75)	0.38	0.0606
North Beach	1	26	6.65 (3.67)	15.19 (4.89)	8.54	0.0002
Financial District/South Beach	1	26	3.35 (2.42)	6.50 (4.31)	3.15	0.0030

^a^ Number (No.) of weeks observed is per six-month period (e.g. in the Golden Gate Park neighborhood, 26 weeks pre-intervention were compared to 26 weeks post-intervention across 2 Pit Stop interventions, resulting in the comparison of 52 weeks pre-intervention versus 52 weeks post-intervention)

^b^ *P-*values are estimated from nonparametric permutation tests (*n* = 10,000 permutations) comparing the difference in the sample means post- versus pre-intervention.

^c^ Neighborhoods with at least one new restroom installed. *SD* Standard deviation

**Fig. 2:**
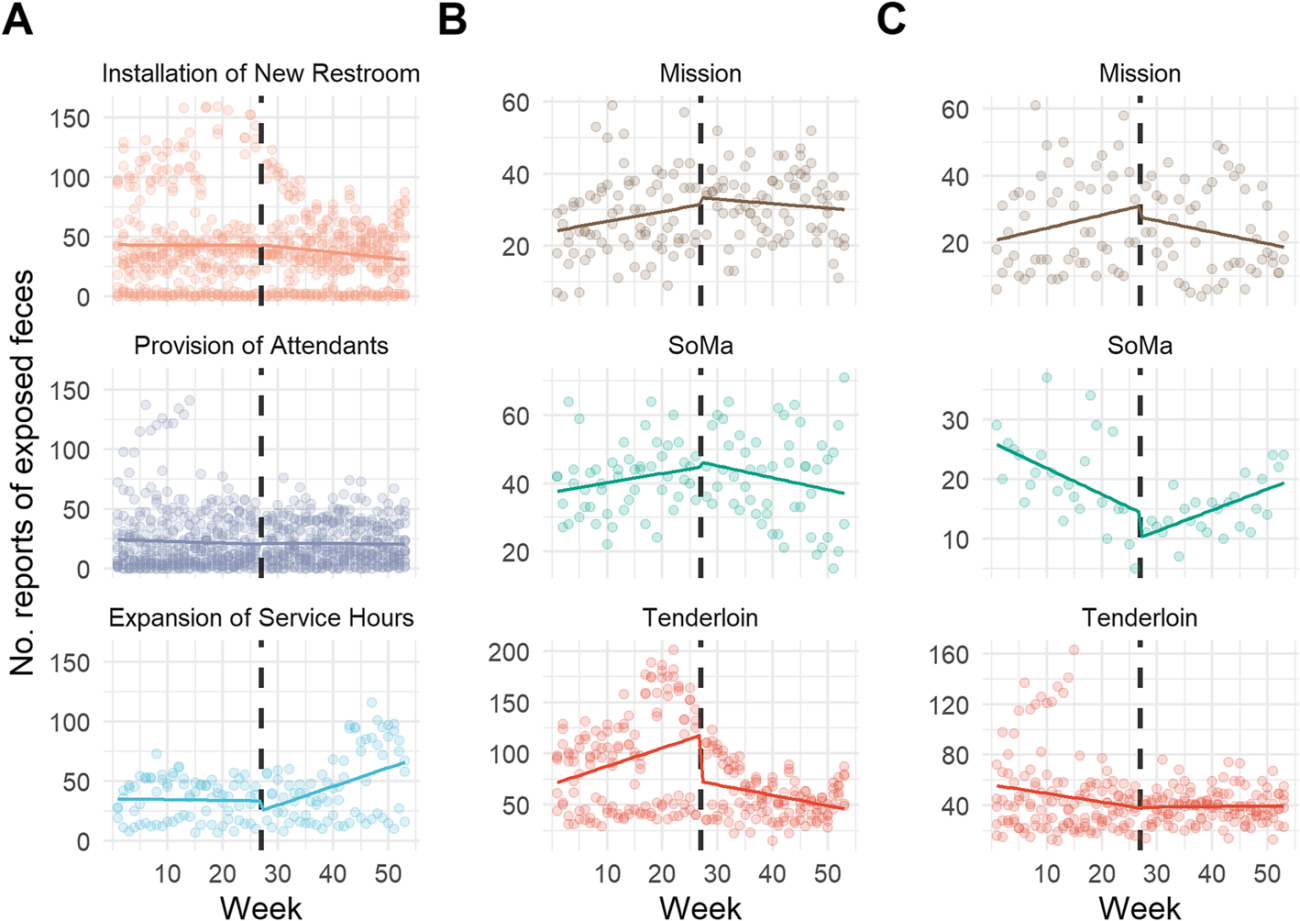
Feces reports by intervention type (**A**) and by neighborhood for new restroom installations (**B**) and the provision of attendants (**C**). Legend: Dashed vertical lines indicate the intervention start date. Individual points represent the number of feces reports per week within a 500 m walking distance buffer of each Pit Stop intervention. Solid horizontal lines represent the slope of weekly feces reports before and after intervention start dates. Only neighborhoods with > 10 feces reports per week on average are included in panels B and C

Results from permutation tests are presented in terms of the change in mean feces reports, denoted Δ. The mean number of feces reports near all newly installed Pit Stop restrooms dropped significantly after their installation (Δ = -12.47; *p* = 0.0002) (Table 1). There was no significant reduction in feces reports near Pit Stop locations where attendants were hired to service the restrooms (Δ = -1.88; *p* = 0.2296). Though there were only three Pit Stop locations that expanded service hours to 24 h per day, there was a significant increase in the mean feces reports per week after the expansion of service hours (Δ = 12.00; *p* = 0.0016). Regression results estimating the post-intervention slope change, denoted Δm, showed there was a significant reduction in the rate of feces reports from the six-month post-intervention period to the pre-intervention period (Δm = -0.024 [95% CI = -0.033, -0.014]) across all locations with new restrooms installed (Table 2, Fig. 2). There was no significant change in the rate of feces reports after the provision of attendants across all locations (Δm = -0.001 [-0.011, 0.008]), while there was an increase in the rate of feces reports following the expansion of service hours (Δm = 0.033 [0.021, 0.044]).

**Table 2:** Pre- versus post-intervention rate of feces reports by intervention type and neighborhood

	No. Pit Stop Interventions	Total No. Weeks Observed ^a^	Pre-Intervention Slope (m) (95% CI) ^b^	Post-Intervention Slope Change (Δm) (95% CI) ^b^
*Intervention Type* ^c^				
Installation of New Restroom	13	676	0.013 (0.006, 0.020)	-0.024 (-0.033, -0.014)
Provision of Attendants	15	780	0.002 (-0.006, 0.010)	-0.001 (-0.011, 0.008)
Expansion of Service Hours	3	156	-0.002 (-0.009, 0.006)	0.033 (0.021, 0.044)
*Neighborhood of Intervention*				
*(Installation of New Restroom, only)*				
Tenderloin	5	260	0.020 (0.008, 0.029)	-0.035 (-0.049, -0.021)
Mission	3	156	0.010 (-0.001, 0.022)	-0.015 (-0.029, -0.0005)
South of Market (SoMa)	2	104	0.007 (-0.002, 0.015)	-0.015 (-0.031, 0.001)
Golden Gate Park	1	52	0.027 (-0.079, 0.133)	-0.182 (-0.316, -0.047)
Haight Ashbury	1	52	-0.054 (-0.117, 0.010)	0.055 (-0.017, 0.128)
Bayview Hunters Point	1	52	-0.015 (-0.042, 0.012)	0.0004 (-0.043, 0.044)
*(Provision of Attendants, only)*				
Tenderloin	5	260	-0.015 (-0.026, -0.005)	0.017 (0.004, 0.030)
Mission	2	104	0.016 (-0.003, 0.034)	-0.031 (-0.055, -0.008)
South of Market (SoMa)	1	52	-0.022 (-0.040, -0.005)	0.046 (0.025, 0.066)
Castro/Upper Market	3	156	0.022 (0.007, 0.037)	-0.022 (-0.043, -0.001)
Golden Gate Park	1	52	0.040 (0.003, 0.078)	-0.040 (-0.010, 0.020)
Sunset/Parkside	1	52	0.054 (-0.041, 0.148)	-0.009 (-0.117, 0.099)
North Beach	1	52	0.030 (0.003, 0.058)	-0.023 (-0.055, 0.009)
Financial District/South Beach	1	52	0.065 (0.032, 0.098)	-0.071 (-0.122, -0.021)

^a^ No. (number) of weeks observed indicates total number of weeks across both the pre- and post-intervention periods (52 weeks total per Pit Stop intervention)

^b^ Estimates are from negative binomial regression models with 95% confidence intervals (CI) calculated from robust standard errors

^c^ Models stratified by intervention include neighborhood as a main effect to adjust for confounding

Pit Stop interventions in the Tenderloin neighborhood had the most feces reports, with a mean of 68.01 reports per week (SD = 45.18) pre-intervention (Table 1). Only Pit Stop interventions located in the Tenderloin resulted in a significant reduction in the mean number of nearby feces reports per week (Δ = -17.60, *p* = 0.0002). There were significant increases in the mean number of feces reports after Pit Stop interventions were implemented in the Castro/Upper Market (Δ = 3.09, *p* = 0.0004), North Beach (Δ = 8.54, *p* = 0.0002), and the Financial District/South Beach (Δ = 3.15, *p* = 0.0030) (Table 1).

Regression results from the interrupted time series analysis identified different changes in long-term trends of feces reports per week when stratified by neighborhood and intervention type. Among Pit Stop locations where new restrooms were installed, the rate of feces reports was significantly lower in the six-months post-intervention compared to the pre-intervention period in the Tenderloin (Δm = -0.035 [-0.049, -0.021]), the Mission (Δm = -0.015 [-0.029, -0.0005]), and Golden Gate Park (Δm = -0.182 [-0.316,-0.047]) (Table 2). The rate of feces reports also declined after new restrooms were installed in SoMa, though the slope change was not statistically significant (Δm = -0.015 [-0.029, 0.001]). Among existing restroom locations where attendants were provided, there were significant reductions in the rate of feces reports near Pit Stops in the Mission (Δm = -0.031 [-0.055, -0.008]), the Castro/Upper Market (Δm = -0.022 [-0.043, -0.001]) and the Financial District/South Beach (Δm = -0.071 [-0.122, -0.021]) (Table 2). The rate of feces reports significantly increased after the provision of attendants at existing Pit Stop locations in the Tenderloin (Δm = 0.017 [0.004, 0.030]) and SoMa (Δm = 0.046 [0.025, 0.066]) (Table 2, Fig. 2).

## Discussion

This study found that the installation of public restrooms as part of the San Francisco Pit Stop program was associated with a long-term reduction in the rate of reports of exposed feces. The decline in feces reports after the installation of new restrooms was driven by reductions in the Tenderloin, the Mission, Golden Gate Park and, to a lesser extent, SoMa. In certain locations, the provision of attendants at existing restrooms led to significant reductions in the rate of feces reports: the Mission, the Castro/Upper Market and the Financial District/South Beach. These results suggest that the addition of new restrooms and the provision of attendants in certain contexts may improve access to and the quality of sanitation facilities, thus reducing open defecation for vulnerable populations without access to other sanitation solutions.

In 2019, the San Francisco point-in-time count of PEH estimated that District 6, which contains the Tenderloin and SoMa neighborhoods, had 3,656 homeless residents, double the amount in the next-highest district (District 10) [4]. Of PEH in District 6, 1,990 (54%) were unsheltered. Unsheltered individuals lack access to the limited shared sanitation facilities offered by homeless shelters and other housing programs and are more likely to have to resort to open defecation [1, 5]. Results from this study suggest that improvements in restroom quality and accessibility have a more appreciable impact in areas where the need for them is higher. The Tenderloin and SoMa had the highest number of feces reports compared to other neighborhoods. This suggests that these neighborhoods may have the highest incidence of open defecation, which aligns with the high prevalence of unsheltered PEH in these areas.

We found that Pit Stop locations in the Tenderloin had the largest average reduction in reports of exposed feces following the interventions. Despite the high number of feces reports and the high prevalence of homelessness in SoMa, there was only a near-significant decline in the post-intervention rate of feces reports near SoMa Pit Stops. We also observed a significant decline in the post-intervention rate of feces reports in the Mission, which had the third highest mean number of reports of exposed feces near Pit Stop locations. According to the 2019 point-in-time count, there were 643 total PEH including 257 (40%) unsheltered PEH in the Mission District neighborhood that is part of District 9 (Fig. 1) [4]. Though the reported population of PEH in the Mission is much lower than in the Tenderloin and SoMa, the estimated total number of PEH in the Mission District is increasing; District 9’s point-in-time count was 410 in 2015 and 552 in 2017 [22, 23]. The Mission also shares its southern and eastern borders with District 10, which had the second highest point-in-time count in 2019 (1,820 PEH) and a single Pit Stop restroom located in Bayview Hunters Point [4]. Notably, there were eleven Pit Stop intervention sites in the Tenderloin. In contrast, there were only four Pit Stop intervention sites within SoMa and five Pit Stop intervention sites in the Mission, which were spread across a large area. Other city-based studies have documented how sanitation coverage can reduce fecal contamination. In a study of low-income urban neighborhoods of Accra, Ghana, increased spatial clustering of sanitation coverage was associated with reduced environmental fecal bacteria contamination [24]. The Tenderloin Pit Stops may have had a greater impact on reducing fecal contamination because more Pit Stop restrooms were clustered together within a smaller area, providing more sanitation facilities within a short walking distance of many PEH. Given the high number of fecal reports observed in SoMa and the Mission, increasing the density of Pit Stop restrooms near known areas with unsheltered people would result in more comprehensive access to sanitation facilities, potentially yielding greater reductions in open defecation.

Despite the promising results after new restroom installations in the Tenderloin, Mission, and SoMa neighborhoods and after the provision of attendants in the Mission, it was unexpected that reports of fecal contamination increased after the provision of attendants and expansion of service hours in some neighborhoods. A 2017 audit of public toilets serving over 3,600 PEH in Los Angeles’ Skid Row neighborhood reported that toilets without attendants had the lowest levels of use (“No Place to Go” 2017, Supplemental Materials, Appendix A). However, this report also noted that the presence of male attendants outside women’s toilets deterred women from using the restroom, especially during overnight hours. Notably, there were only nine toilets for 1,777 people who were unsheltered during the overnight hours in Skid Row, and many of these restrooms had observable fecal matter present, were missing stall doors or had doors that did not lock, and lacked soap, paper towels, seat covers, and menstrual products. Expanding service hours to 24 h per day may increase overnight restroom access for some PEH in San Francisco, though only three restrooms in three different neighborhoods had overnight service hours beginning in 2019 (Supplemental Materials, Table S1). The Sphere standards for sanitation in long-term refugee camps, endorsed by the United Nations High Commission for Refugees, state that there should be at least one toilet for every twenty persons, and that no person should be dwelling further than 50 m from a toilet (The Sphere Handbook 2018, Supplemental Materials, Appendix A). Based on this indicator, there should be 100 toilets available for the 1,990 unsheltered residents of the Tenderloin and SoMa neighborhoods, alone. Restrooms should be distributed throughout these neighborhoods to reduce the distance between any single dwelling and a public toilet. Based on the evidence from Los Angeles, restrooms must be frequently maintained and cleaned and should be staffed appropriately to ensure that all PEH feel safe and comfortable using the restroom, regardless of their gender identity, sexual orientation, race, or ethnicity. A community-engaged and neighborhood-specific audit of the Pit Stop program in San Francisco, similar to the audit conducted in Skid Row of Los Angeles, may be necessary to understand the full scope of the impacts and shortcomings of Pit Stop restrooms as experienced by PEH.

Unsheltered individuals in neighborhoods with poor access to sanitation may be at greater risk of exposure to fecal contamination from open defecation in their surrounding environment. Our results suggest that in San Francisco, unsheltered individuals in the Tenderloin, SoMa, and the Mission are most at risk of exposure to feces based on the high number of 311 exposed feces reports. Feces of humans, as well as dogs, may contain harmful pathogens that pose public health risks to the homeless communities in these neighborhoods. A 2018 study in Atlanta, Georgia detected harmful pathogens in 23% of human fecal samples collected from various open defecation sites [25]. Poor sanitation is a known contributing factor to the spread of infectious diseases in communities worldwide [26]. Additionally, homelessness has been identified as a potential risk factor for antimicrobial resistant infections in San Francisco, CA and elsewhere [8, 27, 28]. PEH are frequent visitors of emergency rooms, often due to mental health needs or substance abuse, increasing their risk of exposure to drug-resistant pathogens that are difficult to treat [29, 30]. Individuals who acquire drug-resistant infections in the hospital may spread drug resistance to others in their community, especially where sanitation and hygiene conditions are inadequate. Future studies should identify pathogens in exposed feces in the urban environment of San Francisco and characterize pathogen carriage among PEH to determine the extent to which exposure to human feces drives infections in these vulnerable communities.

Our analysis has some limitations, the first being the use of 311 Human/Animal Waste reports as a proxy for incidence of open defecation, which may be prone to user error and misclassification. Reports that are correctly classified as Human/Animal Waste may not correspond to a human open defecation event, but may instead be animal feces (especially dog feces), though we were unable to distinguish between reports for human versus animal feces. Animal feces are an important source of exposure to fecal pathogens that can cause diarrheal diseases and other adverse health effects in humans [31]. Pit Stops are equipped with dog waste bags so it is possible that they reduce both human and animal fecal contamination. Further research is warranted to determine the impacts of Pit Stop interventions on reducing animal versus human fecal contamination in San Francisco.

Second, season may have played a role in both the incidence of open defecation and the incidence of reporting exposed feces. In this analysis covering six years of data, reports of feces near Pit Stop intervention sites were highest in the spring and summer months. Seasonal differences in feces reports may have been driven by reports in 2014 and 2017, as the number of reports were more consistent across seasons in 2015, 2016, 2018, and 2019 (Supplemental Materials, Figure S1). Season may also influence the frequency of reporting, as pedestrian traffic may decrease during the colder, rainy months, thereby reducing the chance that someone will encounter and report exposed feces. It is possible that the reduction in reports of feces following the installation of new restrooms in the Tenderloin may be attributable to these seasonal trends. Specifically, after three of the five new Pit Stop restrooms in the Tenderloin were installed on July 15, 2014, there was a citywide reduction in the number of feces reports in all of San Francisco, though a majority of the citywide reports at that time occurred in the Tenderloin (Supplemental Materials, Figure S2).

Third, other time-specific factors, such as changes in public awareness of the presence of feces on sidewalks or the 311 reporting system, may be confounders. Between August and October of 2018, there were at least three events that led to increased media coverage in the San Francisco chronicle and elsewhere: 1) in August, San Francisco DPW announced its plan to create a “Poop Patrol”; 2) in September, an online report about 311 feces reports in San Francisco and other major cities called “Doo-Doo, the New Urban Crisis” was published; and 3) in October, the creation of a free phone app called SnapCrap, designed to make 311 feces reporting in San Francisco more user-friendly, was announced (Supplemental Materials, Appendix A). Media events like these may account for some fluctuations in feces reports throughout the study period. It is possible that there was unmeasured confounding due to changes in public awareness, pedestrian traffic, or misclassification of animal feces as open defecation. While 311 feces reports can be a useful tool to plan and evaluate sanitation interventions, additional research is needed to validate these reports as an accurate and reliable indicator of open defecation over time. Additionally, this study would have benefitted from more detailed audit data including observation data of toilet operations and maintenance, information about the changing roles of restroom attendants at different intervention sites, as well as user experiences.

This study has many strengths, several of which address potential confounding due to seasonal or temporal trends. First, our interrupted time series analysis utilized a multiple baseline design (i.e. interventions beginning on various dates), which inherently controls for time-specific confounding factors. Second, this approach allows for each intervention to serve as its own control during the pre-intervention period, controlling for location-specific factors at each intervention site. Third, we assessed long-term changes in the rate of feces reports per week over a 12-month period, preventing short-term time-specific confounding from biasing our results.

The United Nations General Assembly passed Resolution 64/292 in 2010 (and reaffirmed in 2018) declaring that adequate access to safe water and sanitation are essential human rights [32]. California became the first state to legally recognize the human right to water for drinking, cooking and sanitary purposes with the passage of Assembly Bill 685 in 2012 [33]. However, this bill failed to recognize the human right to access to sanitation, and basic sanitation needs remain unmet in the most vulnerable populations of California. According to the 2019 point-in-time count, there are at least 108,432 unsheltered PEH in the state of California and at least 5,180 unsheltered PEH in the city of San Francisco [4, 34]. The Pit Stop Program improved access to sanitation facilities in San Francisco neighborhoods with the highest number of unsheltered people. This study provides evidence that a public sanitation program can reduce reports of exposed feces in public spaces, especially in neighborhoods with the greatest need for sanitation facilities. Though the Pit Stop Program attempts to fill the gap in sanitation access in San Francisco despite the lack of state legislation to do so, explicitly recognizing basic sanitation as a human right would drive other cities across California to improve sanitation access for all.

## Conclusions

Increased access to public toilets reduced feces reports in San Francisco, especially in neighborhoods with people experiencing homelessness. The addition of restroom attendants also appeared to have reduced feces reports in some neighborhoods with PEH. Based on the findings of this analysis, we recommend that the San Francisco Pit Stop Program be expanded to increase sanitation coverage in SoMa and other areas with high numbers of PEH. Allowing such conditions to persist constitutes a violation of basic human rights and human dignity, and poses a significant public health risk. We also recommend that the city of San Francisco conduct a community-engaged audit of public toilets to understand the full scope of its impacts and shortfalls as experienced by PEH with different gender and racial identities and sexual orientations. It is imperative that state and local governments in California and elsewhere prioritize effective interventions that improve sanitation access, such as San Francisco’s Pit Stop Program, while simultaneously pursuing measures that improve housing affordability and reduce homelessness.

## Supplementary Information

**Additional file 1.**

## Data Availability

Data and code used to conduct this analysis are publicly available online: https://github.com/HeatherKAmato/pit-stop

## Abbreviations

PEH: People Experiencing Homelessness
DPW: Department of Public Works
JMP: Joint Monitoring Program
SD: Standard Deviation
