# Supplementary material for: Somewhere to go: assessing the impact of public restroom interventions on reports of open defecation in San Francisco, California from 2014 to 2020
Source: BMC Public Health. 2022 Sep 4;22:1673. doi: 10.1186/s12889-022-13904-4 (PMC9441075; doi:10.1186/s12889-022-13904-4)
Supplement: Supplementary file 1 — Additional file 1. [file 12889_2022_13904_MOESM1_ESM.docx]

**Supplemental Materials**

**Table S1.** Pit Stop public restroom interventions grouped by neighborhood in San Francisco, California between January 1, 2014 and January 1, 2020.

| Pit Stop Location | Restroom Type | Intervention | Intervention  Start Date |
| --- | --- | --- | --- |
| *Bayview Hunters Point* |  |  |  |
| 3rd St. & Palou Ave. (Mendell Plaza) | Portable | Installation of New Restroom | 7/1/2016 |
| *Castro/Upper Market* |  |  |  |
| Market St. & Castro St. | JC Decaux | Provision of Attendants | 4/6/2016 |
| Market St. & Castro St. | JC Decaux | Expansion of Service Hours | 8/16/2019 |
| Market St. & Church St. | JC Decaux | Provision of Attendants | 6/12/2015 |
| Market St. & Church St.* | JC Decaux | Provision of Attendants | 7/1/2018 |
| *Financial District/South Beach* |  |  |  |
| Embarcadero & Market St. (Embarcadero Plaza) | JC Decaux | Provision of Attendants | 7/1/2018 |
| *Golden Gate Park* |  |  |  |
| Ocean Beach | Portable | Installation of New Restroom | 6/10/2019 |
| Stanyan St. & Waller St. | JC Decaux | Provision of Attendants | 12/5/2015 |
| *Haight Ashbury* |  |  |  |
| Haight St. & Buena Vista Ave. (Buena Vista Park) | Portable | Installation of New Restroom | 6/15/2016 |
| *Mission* |  |  |  |
| 16th St. & Capp St. | Portable | Installation of New Restroom | 6/30/2015 |
| 16th & Mission St. | JC Decaux | Provision of Attendants | 6/30/2015 |
| 24th & Mission St. | JC Decaux | Provision of Attendants | 10/1/2018 |
| McCoppin St. & Valencia St. | Portable | Installation of New Restroom | 6/15/2016 |
| Mission & Sycamore St. | Portable | Installation of New Restroom | 2/1/2016 |
| *North Beach* |  |  |  |
| Washington Square Park | JC Decaux | Provision of Attendants | 7/1/2018 |
| *South of Market (SoMa)* |  |  |  |
| 6th St. & Jessie St. | Portable | Installation of New Restroom | 4/22/2015 |
| 6th St. & Jessie St. | Portable | Expansion of Service Hours | 8/16/2019 |
| 9th St. & Natoma St. | Portable | Installation of New Restroom | 7/1/2016 |
| Victoria Manalo Draves Park | RPD | Provision of Attendants | 1/1/2017 |
| *Sunset/Parkside* |  |  |  |
| Judah St. & La Playa St. | RPD | Provision of Attendants | 7/1/2018 |
| *Tenderloin* |  |  |  |
| 101 Hyde St. | Portable | Installation of New Restroom | 7/15/2014 |
| 133 Golden Gate Ave. | Portable | Installation of New Restroom | 7/15/2014 |
| 30 Grove St. (SF Public Library) | Portable | Installation of New Restroom | 9/12/2016 |
| 388 Ellis St. | Portable | Installation of New Restroom | 7/15/2014 |
| Eddy St. & Jones St. | JC Decaux | Provision of Attendants | 8/1/2017 |
| Eddy St. & Jones St. | JC Decaux | Expansion of Service Hours | 8/16/2019 |
| Eddy St. & Larkin St. | Portable | Installation of New Restroom | 11/14/2018 |
| Grove & Larkin St. (Civic Center Plaza) | JC Decaux | Provision of Attendants | 7/1/2015 |
| 5th St. & Market St. (Hallidie Plaza) | JC Decaux | Provision of Attendants | 9/8/2016 |
| Myrtle & Larkin St. | JC Decaux | Provision of Attendants | 3/5/2018 |
| Market St. & 7th St. (U.N. Plaza) | JC Decaux | Provision of Attendants | 7/1/2015 |

Note: *Pit Stop intervention was implemented temporarily in 2015 and later reintroduced in 2018. RPD = Recreation and Park Department.

**Figure S1.** Temporal trends of exposed feces reports per week within a 500-meter walking distance buffer of Pit Stop interventions implemented in San Francisco, California between January 1, 2014 and January 1, 2020 separated by year and season. Dashed vertical lines indicate the start date of Pit Stop interventions included in the analysis. Mean represents average number of feces reports per week across all Pit Stop locations by year (SD=standard deviation; N=total number of weekly reports across all locations by year).
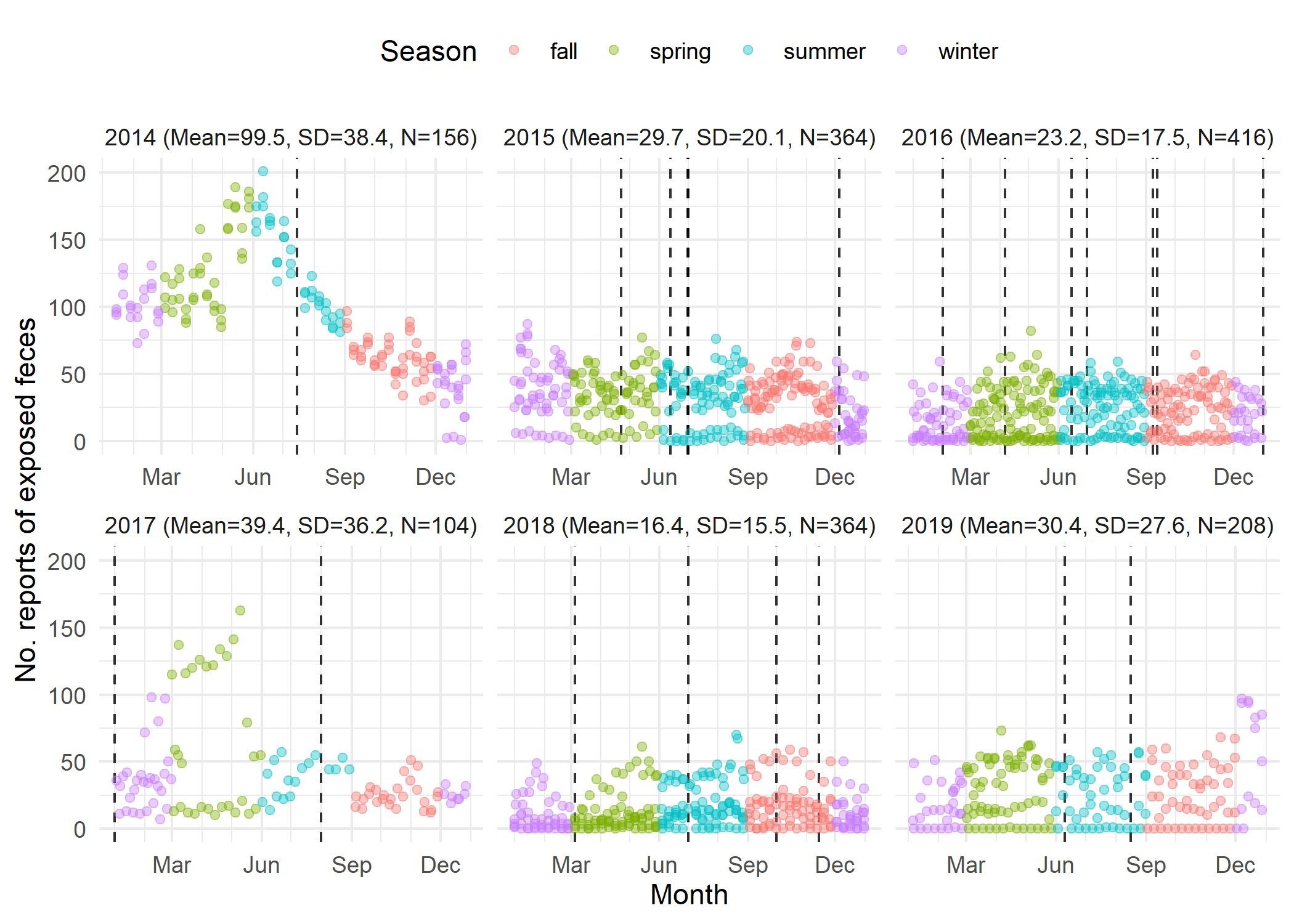


**Figure S2.** Exposed feces reports per week in all of San Francisco, California between 2014 and 2020. Vertical lines indicate Pit Stop intervention start dates (blue=new restroom installation; green=provision of attendants; red=expansion of service hours).
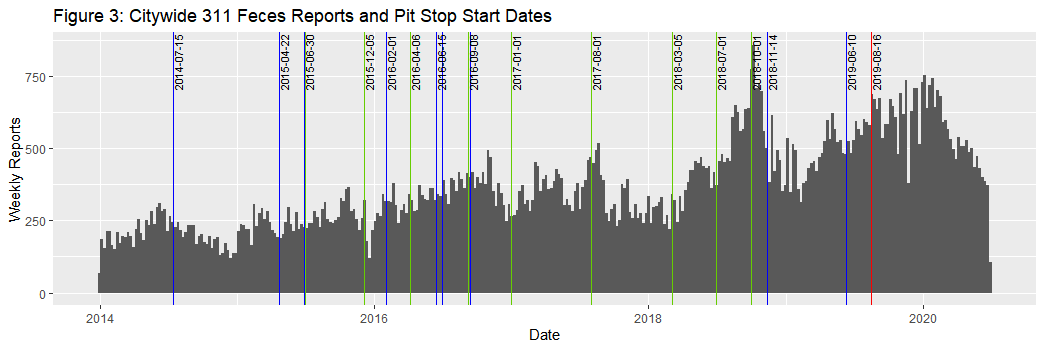


**Appendix A.**

Supplemental References

No Place To Go: An audit of the public toilet crisis in Skid Row. Published June 2017. Accessed March 30, 2022. https://lafla.org/wp-content/uploads/2017/08/No-Place-To-Go-final.pdf

The Sphere Handbook: Humanitarian Charter and Minimum Standards in Humanitarian Response. Fourth edition, Sphere Association, 2018. Accessed March 30, 2022. https://cms.emergency.unhcr.org/documents/11982/32968/Sphere+Handbook+%282018%29/da022ac9-0f23-4ac0-b1a9-b4239cc65456#_ga=2.150147318.1379321749.1649024965-639639887.1649024965

It’s no laughing matter - SF forming Poop Patrol to keep sidewalks clean. SFChronicle.com. Published August 14, 2018. Accessed December 4, 2020. https://www.sfchronicle.com/bayarea/heatherknight/article/It-s-no-laughing-matter-SF-forming-Poop-13153517.php

Doo-Doo, the New Urban Crisis - RealtyHop. Accessed December 4, 2020. https://www.realtyhop.com/blog/doo-doo-the-new-urban-crisis/

Snapcrap - How I built an app to solve San Francisco’s poop problem. Snapcrap - How I built an app to solve San Francisco’s poop problem. Accessed December 4, 2020. https://www.plivo.com/blog/snapcrap-how-built-app-solve-san-francisco-poop-problem/
